# Alterations in mitochondrial energy metabolites following acute subconcussive head impacts among athletes with and without ADHD

**DOI:** 10.1016/j.isci.2025.112776

**Published:** 2025-05-28

**Authors:** Gage Ellis, Madeleine K. Nowak, William G. Kronenberger, Grace O. Recht, Osamudiamen Ogbeide, Lillian M. Klemsz, Patrick D. Quinn, Landon Wilson, Taylor Berryhill, Stephen Barnes, Sharlene D. Newman, Keisuke Kawata

**Affiliations:** 1 Department of Kinesiology, School of Public Health-Bloomington, Indiana University, Bloomington, IN, USA; 2 Program in Neuroscience, College of Arts and Sciences, Indiana University, Bloomington, IN, USA; 3 The Translational Research Center for TBI and Stress Disorders (TRACTS) at VA Boston Healthcare System, Boston, MA, USA; 4 Department of Psychiatry, Boston University Chobanian & Avedisian School of Medicine, Boston, MA, USA; 5 Department of Psychiatry, Indiana University School of Medicine, Indianapolis, IN, USA; 6 Department of Applied Health Science, School of Public Health-Bloomington, Indiana University, Bloomington, IN, USA; 7 Department of Pharmacology and Toxicology, The University of Alabama at Birmingham, Birmingham, AL, USA; 8 Alabama Life Research Institute, University of Alabama, Tuscaloosa, AL, USA; 9 Department of Pediatrics, Indiana University School of Medicine, Indianapolis, IN, USA

**Keywords:** Earth sciences, Environmental science, Land use, Risk assessment

## Abstract

Attention-deficit/hyperactivity disorder (ADHD) is prevalent among contact sports athletes, who may regularly incur repetitive head impacts. This study investigated the effects of acute head impacts on mitochondrial function by analyzing tricarboxylic acid (TCA) cycle metabolites and the potential modulatory role of ADHD. Fifty adult soccer players (ADHD *n* = 25; non-ADHD *n* = 25) participated, undergoing ten soccer headers using a controlled heading model. TCA metabolites were assessed at pre-heading baseline, and 2 and 24 h post-heading. Baseline analysis revealed elevated levels of TCA metabolites, including oxaloacetate, citrate, and isocitrate, in the ADHD group. Following head impacts, both groups exhibited significant decreases in these metabolites, yet the magnitude of decrease was more pronounced in the ADHD group. Pyruvate, alpha-ketoglutarate, and fumarate levels increased after headers in both groups. These findings suggest that ADHD is associated with elevated baseline metabolites initiating the TCA cycle, while acute head impacts induce mitochondrial dysfunction, regardless of ADHD.

## Highlights

• Individuals with ADHD exhibit elevated baseline levels of Krebs cycle metabolites
• Ten headers altered most mitochondrial metabolites regardless of ADHD
• Byproducts of Krebs cycle metabolites were also elevated after ten headers

## Graphical abstract

**Figure:**
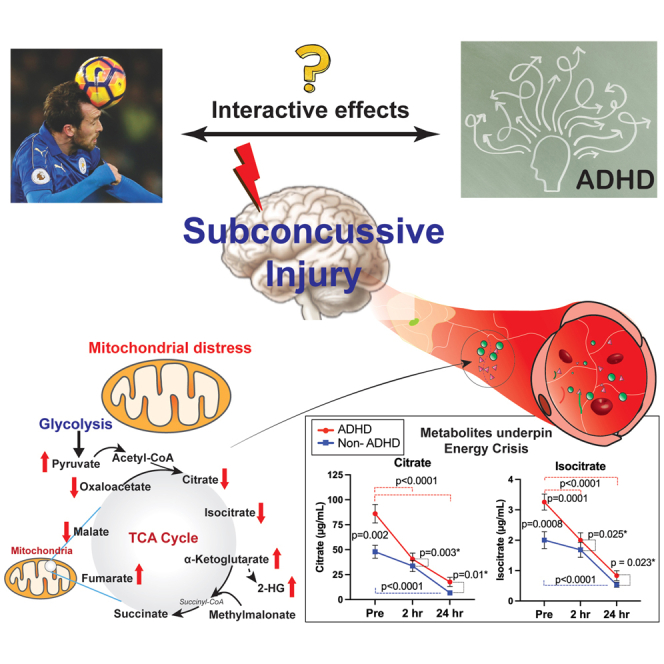

## Introduction

Attention-deficient/hyperactivity disorder (ADHD) is a neurodevelopmental condition with a prevalence rate of 8.1% in children and adolescents and 3.1% in adults.^1^^,^^2^ It is characterized by behavioral symptoms such as inattention, hyperactivity, and impulsivity, which frequently persist into adulthood.^3^^,^^4^ Physical activity, such as recreational sports, has been shown to mitigate symptoms of ADHD^5^; however, participation in contact sports like rugby, American football, and soccer poses the risk of concussion.^6^^,^^7^^,^^8^ Multiple studies have demonstrated that individuals with ADHD are at an increased risk of concussions, exhibit more severe post-concussion symptoms, and experience prolonged recovery period.^7^^,^^8^^,^^9^ Recent investigations further suggest that ADHD may heighten susceptibility to repetitive subconcussive head impacts. This is evidenced by acute declines in memory performance and increased levels of brain-injury blood biomarkers (UCH-L1, GFAP) after sustaining 10 soccer headings.^9^^,^^10^ These findings are particularly concerning given the higher prevalence of ADHD within athletic populations,^11^ with up to 14% of high school athletes and 10% of college athletes having been diagnosed with ADHD.^8^

The increased susceptibility to head impacts associated with the ADHD diagnosis may partially stem from discrepancies in neurobiological pathway compared to non-ADHD individuals.^4^^,^^12^^,^^13^^,^^14^ To explore this hypothesis, researchers have conducted metabolomic analyses—a method for identifying metabolites, or small bioenergetic molecules, involved in various chemical and physiological processes—to identify molecular artifacts indicative of ADHD’s unique biological state. Metabolomic studies in ADHD populations have revealed disruptions in tyrosine production, a key amino acid for synthesizing dopamine, in children with ADHD.^15^^,^^16^ In addition to reduced tyrosine and dopamine production, individuals with ADHD have been shown to possess fewer dopamine (DA1) receptors in the striatum.^4^^,^^13^ Furthermore, a dysregulation of energy metabolism, specifically in glucose metabolism, has also been observed in the frontal lobes of children with ADHD.^15^^,^^16^ This dysregulation aligns with the cellular energy crisis experienced by concussed individuals. A seminal 2001 paper by Giza and Hovda described how, after brain injury, individuals initially undergo a state of hyperglycolysis followed by a prolonged reduction in glucose metabolism, which can last several weeks in some cases.^17^ More specifically, neuronal cells are burdened by the overconsumption of energy sources (adenosine triphosphate or ATP) by cell membrane ionic pumps during hyperglycolysis, destabilizing energy balance. Simultaneously, due to excessive calcium influx into mitochondria, ATP production is attenuated.^17^^,^^18^ When the energy cycle is disrupted, subsequent injuries can destabilize the system, leading to long-lasting impairments.^17^^,^^18^

In addition to the mitochondrial metabolic dysregulation induced by concussions, emerging evidence suggests that repetitive subconcussive head impacts may elicit similar cellular responses. Vike et al.^19^^,^^20^ conducted a study involving 23 male collegiate American football players, measuring their plasma levels of metabolites associated with glycolytic, gluconeogenesis, and pyruvate metabolism pathways both pre-season baseline and post-season. Most notable post-season changes were observed in the tricarboxylic acid (TCA) cycle, which plays a crucial role in supplying energy metabolites to mitochondrial electron transport chains for ATP production. Significant reductions in key metabolites, including citrate, alpha-ketogluterate, fumarate, and malate, were detected, suggesting that season-long exposure to repetitive head impacts may impair cellular energy production and contribute to metabolic crisis.^19^ While these findings provide a novel translation of preclinical insights into clinical settings, their interpretation is limited by uncontrolled extraneous variables (e.g., physical exhaustion, exercise, and bodily damage), which complicate the isolation of head impact effects. Additionally, variability in head impact exposure across players further confounds the results. Moreover, it remains unclear whether neuronal metabolic responses to subconcussive head impacts are more pronounced in individuals with ADHD compared to athletes without ADHD, or whether no significant differences exist between the two groups.

To address these limitations and enhance the generalizability of previous research, we conducted a case-control intervention study to examine plasma levels of metabolites related to mitochondrial TCA cycle following acute subconcussive head impacts in individuals with and without ADHD diagnosis. We used our established soccer heading model^21^ to induce 10 controlled subconcussive head impacts while eliminating extraneous influences that are inherent in field studies, including bodily hits, fatigue, exercise, and perspiration/hydration. Since individuals with ADHD have been shown to respond adversely to concussive and subconcussive head impacts, we hypothesized that while both groups would exhibit alterations in mitochondrial energy metabolites following headers, changes would be amplified in the ADHD group compared to the non-ADHD group.

## Results

### Demographics

**Figure 1:**
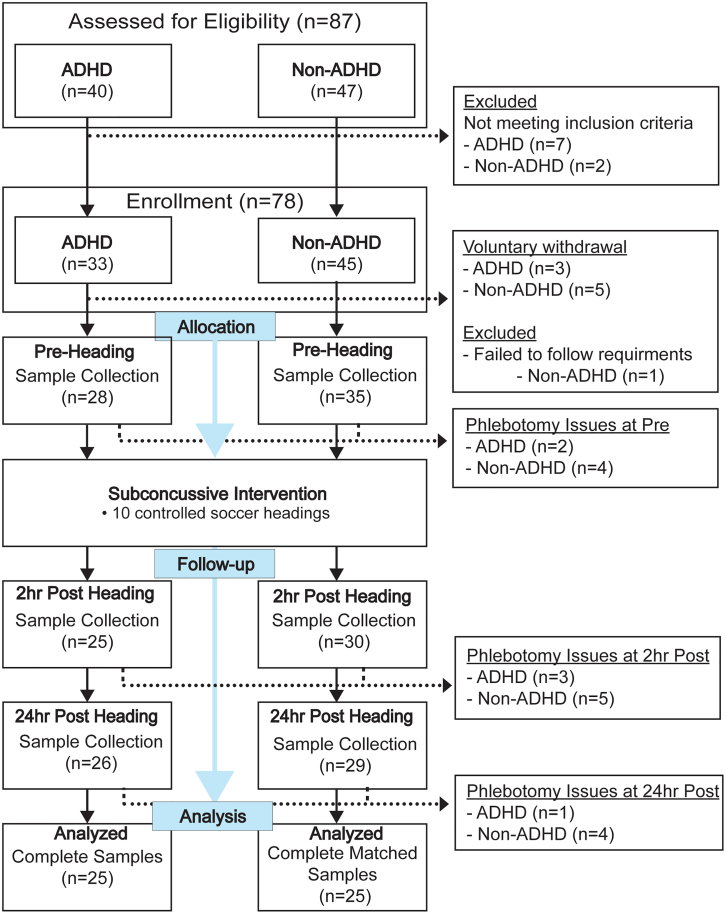
Study flow chart

Eighty-seven individuals were assessed for eligibility, and 78 individuals who met the inclusion criteria and were free of exclusion criteria proceeded to the study. Changes in schedules and COVID-19 exposure/diagnosis led to eight voluntary withdrawals prior to the study data collection. One additional individual withdrew due to failure to comply with study instructions. As a result, a total of 69 participants completed the study. Venous blood samples were not obtained from five participants due to participant anxiety or phlebotomy difficulty. Based on the available sampling pool, 50 pairs of age- and sex-matched participants (ADHD = 25, non-ADHD = 25) were included in TCA intermediate analysis. The study flow chart is depicted in Figure 1. Significant group differences in number of previous concussion and diagnosis of additional mental health disorder (anxiety, depression, or combination of both) were observed and were accounted for in our statistical models. Demographics and head impact kinematics are presented in Table 1.

**Table 1:** Demographics

Variables	ADHD	Non-ADHD
*n*	25	25
Sex	12 M; 13 F	12 M; 13 F
Age, *y*	19.8 ± 2.75	20.44 ± 1.92
BMI, *kg/m*^*2*^	22.37 ± 3.70	23.7 ± 3.23
Soccer Experience, *y*	11.04 ± 3.75	12.64 ± 3.24
Soccer Heading Experience, *y*	7.72 ± 2.51	9.52 ± 3.44

Race, n (%)		
White	20 (80)	20 (80)
Black/African American	2 (8)	1 (4)
Asian	1 (4)	3 (12)
More than one race	2 (8)	1 (4)
ADHD Medication, *days per week*	6.4 ± 0.84	–

**Additional mental health diagnoses ∗∗∗**		
None, *n (%)*	12 (48)	23 (92)
Anxiety, *n (%)*	4 (16)	1 (4)
Depression, *n (%)*	1 (4)	1 (4)
Anxiety and Depression, *n (%)*	8 (32)	0 (0)

**No. Previous Concussion∗∗**		
0, *n (%)*	13 (52)	21 (84)
1, *n (%)*	9 (36)	4 (16)
2, *n (%)*	3 (12)	0 (0)

**Head Impact Kinematics**		
PLA, *g*	13.16 ± 2.10	14.30 ± 2.45
PRA, °*/s2*	826.57 ± 185.67	805.18 ± 205.21

PLA, peak linear acceleration; PRA, peak rotational acceleration; BMI, body mass index; ADHD, attention-deficit/hyperactivity disorder. Asterisk (∗) refers to statistically significant differences between groups at baseline: ∗*p* < 0.05; ∗∗*p* < 0.01; ∗∗∗*p* < 0.001.

### Baseline group differences in TCA-cycle related metabolites

**Figure 2:**
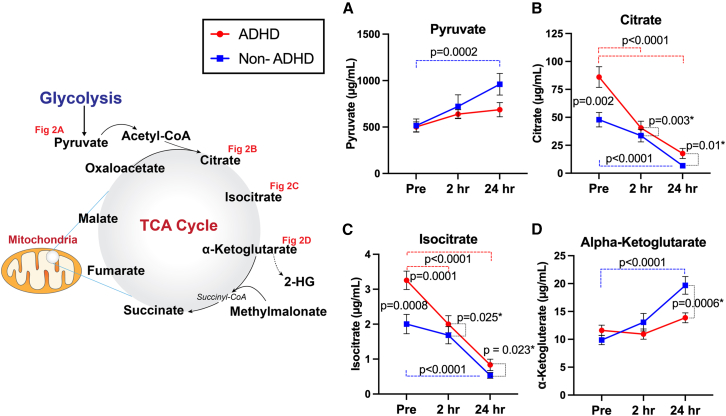
Head impact effects on mitochondria-related metabolites, including pyruvate, citrate, isocitrate, and alpha-ketoglutarate (A–D) The non-ADHD group displayed significant elevations in pyruvate and alpha-ketoglutarate at 24 hours-post heading, while the ADHD group remain stable across time points. Significantly elevated levels of citrate and isocitrate were observed in the ADHD group at baseline, then sharply decreased after head impacts. Lesser degrees of decreases in citrate and isocitrate were observed in the non-ADHD group. ∗indicating results with significant group by time interactions, relative to baseline. Data are represented as mean ± SEM.

**Figure 3:**
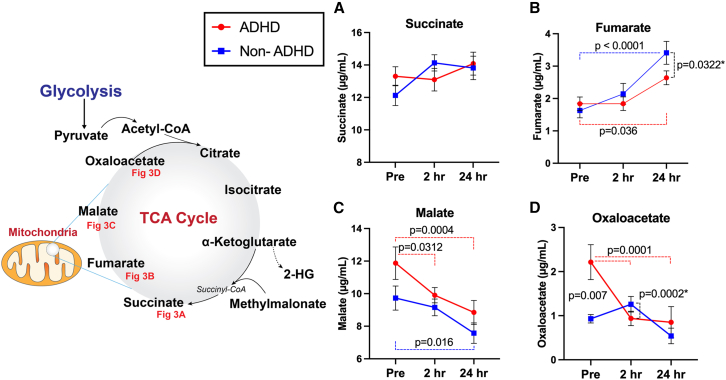
Head impact effects on mitochondria-related metabolites, including succinate, fumarate, malate, and oxaloacetate (A–D) Both groups exhibited increase in fumarate at 24 hours-post heading, yet the degree of increase was slightly greater in the non-ADHD group. Conversely, malate levels showed significant decrease after head impacts in both groups. The ADHD group had decreases in oxaloacetate after 10 soccer headers. The lower baseline oxaloacetate levels in the non-ADHD group coupled with non-significant elevations at 2 hours-post contributed to a statistically significant group-by-time interaction at 2 hours-post heading. ∗indicating results with significant group-by-time interactions, relative to baseline. Data are represented as mean ± SEM.

The ADHD group exhibited significantly elevated pre-heading baseline levels in citrate (36.56 μg/mL, CI[18.55, 54.57], *p* = 0.0002: Figure 2B), isocitrate (1.18 μg/mL, CI[0.52, 1.84], *p* = 0.0008: Figure 2C), malate (2.57 μg/mL, CI[0.43, 4.71], *p* = 0.023: Figure 3C), and oxaloacetate (1.08 μg/mL, CI[0.33, 1.82], *p* = 0.007: Figure 3D) compared to those of the non-ADHD group, whereas other metabolites, including pyruvate, alpha-ketoglutarate, succinate, fumarate, 2-HG, and methylmalonate, did not have statistically significant group differences at baseline. Sex (male vs. female) did not have any significant influence on the outcomes.

### Subconcussive effects on TCA-cycle-related metabolites

Following 10 acute soccer headers, we observed significant changes in the TCA-cycle related metabolites in both groups. First, serum levels of pyruvate increased at 24 hours-post heading in the non-ADHD group (445.0 μg/mL, CI[186.9, 702.0], *p* = 0.0002), whereas pyruvate levels maintained throughout the time points in the ADHD group. Citrate and isocitrate demonstrated near identical patterns, where significant decreases were notable in both groups at 2 h and 24 hours-post heading compared to pre-heading baseline (see Table 2 for within-group changes). However, the magnitude of decrease was more pronounced in the ADHD group, as illustrated by a significant group-by-time interaction (citrate: 2 hours-post, −31.34 μg/mL, CI[−51.43, −11.25], *p* = 0.003, 24 hours-post, −27.26 μg/mL, CI[−47.34, −7.17], *p* = 0.0099; isocitrate: 2 hours-post, −0.93 μg/mL, CI[−1.73, −0.14], *p* = 0.02, 24 hours-post, −0.95 μg/mL, CI[−1.75, −0.15], *p* = 0.02): Figure 2.

**Table 2:** Changes in metabolite level relative to pre-heading baseline

Metabolites	Group	T22-Hours Post-Heading	T324-Hours Post-Heading
Pyruvate (μg/mL)	ADHD	136(−121.4, 394.0)*p* = 0.421	184(−73.4, 442)*p* = 0.209
	Control	205(−52.3, 463.0)*p* = 0.145	445(186.9, 702.0)*p* = 0.0002
Citrate (μg/mL)	ADHD	−45.6(−63.0, −28.15)*p* < 0.0001	−68.4(−85.9, −50.99)*p* < 0.0001
	Control	−14.2(−31.7, 3.19)*p* = 0.132	−41.2(−58.6, −23.74)*p* < 0.0001
Isocitrate (μg/mL)	ADHD	−1.26(−1.95, −0.56)*p* = 0.0001	−2.42(−3.11, −1.73)*p* < 0.0001
	Control	−0.32(−1.01, 0.37)*p* = 0.517	−1.27(−2.16, 0.76)*p* < 0.0001
Alpha-Ketoglutarate (μg/mL)	ADHD	−0.67(−4.26, 2.92)*p* = 0.896	2.26(−1.22, 5.85)*p* = 0.296
	Control	3.18(−0.41, 6.77)*p* = 0.093	9.80(6.21, 13.39)*p* < 0.0001
Succinate (μg/mL)	ADHD	−0.21(−2.19, 1.77)*p* = 0.965	0.78(−1.21, 2.76)*p* = 0.622
	Control	2.00(0.02, 3.99)*p* = 0.047	1.69(−0.29, 3.68)*p* = 0.110
Fumarate (μg/mL)	ADHD	0.0004(−0.76, 0.76)*p* = 1.00	0.80(0.04, 1.56)*p* = 0.036
	Control	0.51(−0.25, 1.27)*p* = 0.247	1.78(1.02, 2.54)*p* < 0.0001
Malate (μg/mL)	ADHD	−1.97(−3.79, −0.14)*p* = 0.031	−3.02(−4.85, −1.20)*p* = 0.0004
	Control	−0.57(−2.40, 1.25)*p* = 0.735	−2.15(−3.97, −0.33)*p* = 0.016
Oxaloacetate (μg/mL)	ADHD	−1.18(−1.85, −0.52)*p* = 0.0001	−1.24(−1.90, −0.58)*p* = 0.0001
	Control	0.31(−0.35, 0.97)*p* = 0.498	−0.38(−1.04, 0.28)*p* = 0.356
2-HG (μg/mL)	ADHD	0.13(−0.33, 0.59)*p* = 0.792	0.50(0.03, 0.96)*p* = 0.031
	Control	0.29(−0.16, 0.75)*p* = 0.282	1.09(0.63, 1.55)*p* < 0.0001
Methylmalonate (μg/mL)	ADHD	−0.02(−0.23, 0.18)*p* = 0.970	0.16(−0.05, 0.37)*p* = 0.165
	Control	0.09(−0.11, 0.30)*p* = 0.519	0.46(0.25, 0.66)*p* < 0.0001

Values are expressed as difference (95% confidence interval) *p* value.

Contrary to the decrease observed in citrate and isocitrate, two succeeding metabolites in the TCA cycle, alpha-ketoglutarate, and fumarate, exhibited significant increase following 10 soccer headers. Specifically, significant increase in alpha-ketogluterate was notable in the non-ADHD group especially at 24 hours-post heading compared to baseline (9.80 μg/mL, CI[6.21, 13.39], *p* < 0.0001), whereas no significant changes were observed overtime in the ADHD group. This resulted in a statistically significant group-by-time interaction at 24 hours-post heading (−7.54 μg/mL, CI[−11.67, 3.41], *p* = 0.0006: Figure 2D). A similar degree of acute elevations in fumarate was observed in both groups at 2 hours- and 24 hours-post heading compared to baseline (Table 2 for detailed statistical output).

The remaining two metabolites within the TCA cycles showed a general trend of decrease after 10 headers in both groups at similar degree. Acute 10 soccer headers led to a significant decrease in malate at both post-heading time points in the ADHD group (2 hours-post: −1.97 μg/mL, CI[−3.79, −0.14], *p* = 0.031; 24 hours-post: −3.02 μg/mL, CI[−4.85, −1.20], *p* = 0.0004), as well as the non-ADHD group (24 hours-post: −2.15 μg/mL, CI[−3.97, −0.33], *p* = 0.0164). Similarly, the ADHD group had a linear decrease in oxaloacetate after 10 soccer headers (2 h: −1.18 μg/mL, CI[−1.85, −0.52], *p* = 0.0001; 24 h: −1.24 μg/mL, CI[−1.90, −0.58], *p* = 0.0001: Figure 3). Of note, while there were no significant within-group changes in oxaloacetate in the non-ADHD group, the lower baseline oxaloacetate levels in the non-ADHD group coupled with non-significant elevations at 2 hours-post contributed to a statistically significant group-by-time interaction at 2 hours-post heading (−1.50 μg/mL, CI[−2.26, −0.74], *p* = 0.0002). Sex (male vs. female) did not have any significant influence on the outcomes. Average values of each metabolite at each time point are detailed in Table S1.

### Subconcussive effects on byproduct metabolites of the TCA cycle

**Figure 4:**
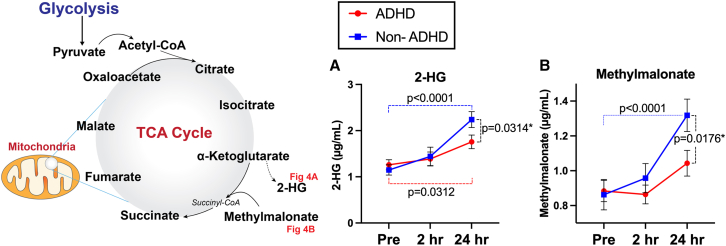
Head impact effects on byproduct metabolites for the TCA cycle (A and B) There were general trends of increase after head impacts in both groups. However, significant acute elevations in 2-HG (A) and methylmalonate (B) were pronounced in the non-ADHD group especially at 24 hours-post heading. For both 2-HG and methylmalonate, significant group-by-time interactions were observed at 24 hours-post heading. ∗indicating results with significant group by time interactions, relative to baseline. Data are represented as mean ± SEM.

In addition to eight TCA-cycle-related metabolites, we explored two additional metabolites (2-hydroxyglutarate and methylmalonate) that are byproducts of the TCA cycle. There were general trends of increase after 10 headers, but the magnitude of increase was pronounced in the non-ADHD group. Specifically, significant acute elevations in 2-HG and methylmalonate occurred at 24 hours-post heading in the non-ADHD group compared to baseline (2-HG: 1.09 μg/mL, CI[0.63, 1.55], *p* < 0.0001; methylmalonate: 0.46 μg/mL, CI[0.25, 0.66], *p* < 0.0001). Slight, but statistically significant elevation in 2-HG was observed in the ADHD group at 24 hours-post heading (0.50 μg/mL, CI[0.03, 0.96], *p* = 0.031). For both 2-HG and methylmalonate, significant group-by-time interactions were observed at 24 hours-post heading, with the non-ADHD group exhibiting a greater increase than the ADHD group (2-HG: −0.60 μg/mL, CI[−1.12, −0.07], *p* = 0.031; methylmalonate: −0.30 μg/mL, CI[−0.54, −0.06], *p* = 0.018: Table 2; Figure 4).

## Discussion

This study provides critical insights by translating preclinical research on the effects of brain injury on cellular energy metabolism into clinical implications. It also expands our knowledge of differential mitochondrial responses to subconcussive head impacts in individuals with and without ADHD. The study revealed three key findings. First, at baseline, individuals with ADHD exhibited elevated levels of key TCA cycle metabolites, including citrate, isocitrate, malate, and oxaloacetate, suggesting heightened resting-state energy metabolism compared to non-ADHD counterparts. Second, independent of ADHD status, ten acute subconcussive head impacts led to significant alterations in most TCA cycle metabolites. These changes displayed a bifurcated pattern: decrease in citrate, isocitrate, malate, and oxaloacetate, alongside increases in pyruvate, alpha-ketoglutarate, succinate, and fumarate. Additionally, byproducts of the TCA cycle, such as 2-HG and methylmalonate, were markedly elevated after ten headers. Third and lastly, while the directional changes in metabolites were consistent between the ADHD and non-ADHD groups, notable differences in magnitude were observed. Specifically, the ADHD group exhibited a greater reduction in citrate and isocitrate, whereas the non-ADHD group demonstrated a more pronounced increase in alpha-ketoglutarate and fumarate. Taken together, these findings suggest that even a brief exposure to subconcussive head impacts may alter mitochondrial energy-related metabolites detectable in blood. While interpreting the role of ADHD remains complex, we discuss potential mechanistic factors within the ADHD group to contextualize our findings.

### Greater cellular metabolic demand in the brains of athletes with ADHD

The role of mitochondrial dysfunction in the pathophysiology of ADHD represents an emerging and rapidly advancing field of research. While the etiology of ADHD remains unclear, increased oxidative stress has been implicated as a critical factor that induces mitochondrial dysfunction. For example, a study conducted by Öğütlü et al.^22^ identified a 1.3-fold increase in mitochondrial DNA (mtDNA) copy number in children with ADHD compared to their non-ADHD counterparts. Similar findings have been reported in other studies, demonstrating not only elevated mtDNA copy numbers^23^ but also associations between the 10398A/G mtDNA polymorphism and aggressive behavior.^24^ Furthermore, increased levels of mitochondrial proteins, including HtrA2, α-synuclein, and Park7, were observed in ADHD patients,^25^ alongside a marked oxidative imbalance characterized by elevated nitric oxide (an oxidant) and reduced superoxide dismutase (an antioxidant).^26^^,^^27^ These findings suggest that mitochondrial dysfunction, mediated by oxidative stress, may play a pivotal role in the pathophysiology of ADHD. In our study, baseline assessments revealed that individuals with ADHD exhibited 1.2- to 2.4-fold higher levels of several TCA cycle metabolites, including citrate, isocitrate, malate, and oxaloacetate. These baseline elevations may reflect a compensatory mechanism aimed at counteracting mitochondrial dysfunction. Alternatively, they could result from increased cellular ATP demands in the brain, given that a meta-analysis of 55 ADHD fMRI task-based studies reported that hyperactivity in the default-mode network in ADHD as one of the most prominent and reliable features in individuals with ADHD.^28^ It is generally noted that levels of citrate and oxaloacetate are indicative of the “energy status” of a cell. Lower levels of citrate and oxaloacetate reflect a reduced energy production, whereas greater levels of these metabolites are associated with increased energy demand.^29^^,^^30^ While our data are consistent with prior ADHD research, it is important to note that baseline metabolite levels in blood may be significantly influenced by extracranial sources, warranting cautious interpretation. In contrast, head impact effects are evaluated using a repeated-measures design with a controlled head impact model, which enables to provide mechanistic relationships between head impacts and brain mitochondrial metabolite responses.

### Head impact effects on TCA cycle metabolites

Our study yielded various noteworthy findings. First, increased levels of pyruvate were observed following head impacts in the non-ADHD group. This observation aligns with the well-established concept that, following traumatic brain injury (TBI), the brain relies on the glycolytic pathway to upregulate pyruvate and meet its heightened energy demand.^17^^,^^18^ Specifically, pyruvate serves as a precursor to the TCA cycle. Through glycolysis, glucose is metabolized into pyruvate, which is subsequently converted by the pyruvate dehydrogenase complex into acetyl-coenzyme A (acetyl-CoA). Acetyl-CoA then combines with oxaloacetate to form citrate, thereby initiating the TCA cycle. Contrary to healthy state, we observed that although pyruvate is elevated after head impacts, citrate and isocitrate (initial metabolites of the TCA cycle), as well as the last metabolite (oxaloacetate), were significantly decreased after head impacts. This may be due to the potential reductions in the activity of pyruvate dehydrogenase.^31^ For example, Sharma et al.^32^ induced closed head injuries in rats using a fluid percussion model and observed a significant reduction in pyruvate dehydrogenase enzyme levels in the blood, accompanied by global oxidative stress and increased gliosis in brain tissue 72 h post-injury. These findings were supported by Lazzarino et al.,^33^ who demonstrated a significant time course reduction in acetyl-CoA levels within brain tissue homogenates of rats with mild TBI over a 120-h period. Collectively, these results suggest that the increased energy demand caused by subconcussive stress accelerates glycolysis; however, the elevated pyruvate flux may not be efficiently converted to acetyl-CoA. This inefficiency could contribute to the depletion of key initiating metabolites in the TCA cycle, such as citrate, isocitrate, and oxaloacetate.

As previously discussed, the ADHD group exhibited significantly higher baseline levels of citrate, isocitrate, malate, and oxaloacetate compared to their non-ADHD counterparts. Following soccer headers, levels of these four metabolites decreased in both groups, with the ADHD group showing a significantly greater magnitude of reduction. Although the clinical implications of these findings remain unclear, the decline in the metabolites critical for initiating the TCA cycle may contribute to heightened cognitive deficits after subconcussive injury,^9^ as well as greater incidence rate, exacerbated symptoms, and prolonged recovery in individuals with ADHD following concussions.^7^^,^^8^^,^^9^

We observed several TCA cycle metabolites displaying contradictory patterns. While citrate and isocitrate demonstrated similar acute declines following soccer headers, subsequent metabolites, including alpha-ketoglutarate, succinate, and fumarate, exhibited increased levels 24 hours-post heading, particularly in the non-ADHD group. This may be attributed to the dual role of alpha-ketoglutarate: not only is it a key intermediate in the TCA cycle, but it also serves as a precursor for glutamate synthesis. Brain trauma, including repetitive subconcussive head impacts,^34^ is known to induce glutamate excitotoxicity, characterized by an excess accumulation of glutamate in the synaptic cleft, leading to hyperactivation of postsynaptic neurons.^17^ The upregulation of alpha-ketoglutarate is a prerequisite for glutamate production in neurons. Alternatively, alpha-ketoglutarate may function as an antioxidant, directly reacting with hydrogen peroxide to mitigate oxidative stress.^35^ This dual role highlights its potential involvement in the metabolic response to subconcussive head impacts. The observed increase in alpha-ketoglutarate likely triggers a cascade effect by elevating the levels of subsequent TCA-cycle metabolites, such as succinate and fumarate. Consequently, a bifurcated metabolic pattern emerged, with significant decreases in citrate, isocitrate, malate, and oxaloacetate, contrasting with increases in alpha-ketoglutarate, succinate, and fumarate, indicative of metabolic dysregulation. A key question remains: why were the upregulations of alpha-ketoglutarate, succinate, and fumarate more pronounced in the non-ADHD group than in the ADHD group? One possible explanation lies in the influence of ADHD medication, warranting further discussions.

### Role of ADHD medication in cellular metabolites

Understanding the neurobiological interaction between ADHD and subconcussive head impacts is influenced by multiple factors, including previous concussion history, ADHD onset, and the diversity of treatment approaches (e.g., behavioral interventions, and pharmaceutical therapies). Among these, literature exploring the role of psychostimulant medication presents a spectrum of outcomes, highlighting both potential benefits and limitations. For example, rodent models exhibiting heightened ADHD-related behaviors demonstrated dysregulated dopamine expression in frontal brain regions and a reduced density of dopamine receptors in the hippocampus, correlating with hyperactive behaviors and cognitive deficits.^36^^,^^37^ Studies on psychostimulant administration in dopamine transporter knockout mice have shown increased dopamine expression in the prefrontal cortex and inhibition of hyperactivity behavior, shedding light on the paradoxical effects of psychostimulants in ADHD management.^38^^,^^39^^,^^40^ Clinically, emerging evidence suggests that psychostimulant medication for ADHD may exert neuroprotective effects in the context of concussions. A 2023 prospective study on concussed athletes with ADHD revealed that those on psychostimulant medication experienced significantly faster recovery times compared to their unmedicated counterparts, despite the ADHD group being at a higher overall risk of concussion.^41^ Similarly, a study by Ali et al.^42^ found that youth athletes with ADHD who used psychostimulant medication had a notably lower risk of concussion (odds ratio 0.51) compared to those not on medication. Furthermore, in a large cohort comprised Swedish and U.S. pediatric population, Ghirardi et al.^43^ revealed that children and adolescents with ADHD (*n* = 9421) had significantly lower incidence of TBI (hazard ratio 0.27) during mediated periods, compared to non-medicated periods. This suggests that ADHD medication not only has potent effects in reducing ADHD-related symptoms but also provides prophylactic measures against TBI.

Conversely, an opposing line of research suggests that ADHD medications, such as methylphenidate (trade name: Ritalin) and atomoxetine (trade name: Stratterra), may contribute to mitochondrial dysfunction. For example, chronic high-dose exposure to methylphenidate (2-10mg/kg) has been shown to decrease the activities of citrate synthase and isocitrate dehydrogenase in young and adult animal models.^44^ These enzymes are crucial for the TCA cycle, condensing acetyl-CoA with oxaloacetate to form citrate and decarboxylating isocitrate to form alpha-ketoglutarate.^45^ A reduction in these enzymatic activities implies an imbalance in the TCA cycle, which could impair brain bioenergetics and provoke cellular adaptations to meet energy demand. Similarly, atomoxetine, a non-stimulant and selective norepinephrine reuptake inhibitor, has demonstrated adverse effects *in vitro* at high concentrations, including increased oxidative stress, mitochondrial mass, and altered mitochondrial membrane potential.^46^ It is important to emphasize that these mitochondrial effects were observed under conditions of high-dose, chronic treatment. While the sample size in our study precludes stratification by medication type and dosage, prescriptions were individualized by their medical providers. Also, a review of participants’ treatment regimens revealed no instances of high-dose medication use, with most individuals being on Adderall, Vyvanse, or Amphetamine Salts. Thus, the role of ADHD medication in our findings remains an open question. Interestingly, despite exhibiting elevated baseline levels of TCA cycle metabolites, the ADHD group showed milder alterations in metabolite levels after acute soccer headers compared to the non-ADHD group, which aligns with findings from previous concussion studies.^41^^,^^42^ To determine whether ADHD medication provides neuroprophylaxis against subconcussive head impacts, future research should include a cohort of individuals with ADHD who are not on medication. Additionally, increasing the number of head impacts (e.g., 15–20 headers) may help elucidate the effects of psychostimulants on cellular metabolic responses.

### Byproduct metabolites of the TCA cycle

In addition to evaluating TCA metabolites, we explored two byproducts of the TCA cycle:2-HG and methylmalonate. Similar to the findings of Vike et al.^19^ who reported a significant increase in 2-HG levels after a college football season compared to preseason baseline, our study observed elevated 2-HG at the 24-h time point in both groups. 2-HG is derived from alpha-ketoglutarate through altered isocitrate dehydrogenase activity or elevated concentration of lactate dehydrogenase or malate dehydrogenase.^47^ Elevated levels of 2-HG have been associated with increased oxidative stress and potential cellular damage.^48^^,^^49^^,^^50^ Methylmalonate levels, on the other hand, were increased only in the non-ADHD group at 24 hours-post heading. Methylmalonate plays a key role in the synthesis of succinyl-CoA from alpha-ketoglutarate and then converted into succinate. Increase in methylmalonate is often indicative of either a TCA cycle imbalance or vitamin B12 deficiency, wherein methylmalonyl-CoA cannot be effectively converted to succinyl-CoA, leading to accumulation of methylmalonate.^51^^,^^52^ Both 2-HG and methylmalonate are considered toxic metabolites at high concentration due to their association with metabolic dysregulation and oxidative stress.

In summary, the data from the current study suggests that individuals with ADHD exhibit significantly elevated baseline levels of key TCA cycle-initiating metabolites, including oxaloacetate, citrate, isocitrate, and malate, compared to their non-ADHD counterparts. Following subconcussive head impacts, mitochondrial dysfunction was evident in both groups, as illustrated by marked alterations, either elevations or decreases, in most TCA cycle metabolites. Metabolites that were elevated at baseline in the ADHD group experienced more pronounced decreases post-heading compared to the non-ADHD group, suggesting a heightened metabolic response to head impacts in ADHD. Additionally, elevations in downstream metabolites, such as alpha ketoglutarate, may imply a potential link to glutamate excitotoxicity. Further research is needed to understand the role of ADHD psychostimulant medication, as well as involvements of non-TCA cycle metabolites (e.g., fatty acids, amino acids, and endocannabinoids), in modulating cellular metabolic responses to subconcussive head impacts in individuals with and without ADHD.

### Limitations of the study

There are several limitations that needed to be accounted for when interpreting the results of this study. First, the sample size was limited to 50 participants. While the presence and severity of ADHD were rigorously assessed using the ADHD investigator symptom rating scale (AISRS), and the control group was matched for age and sex, a larger study is required to enable exploratory stratification analyses on the effects of medication. Additionally, participants were not monitored between the 2- hour and 24-h visits. However, the repeated-measures study design combined with the use of a mixed-effects regression model likely mitigates the impact of extraneous factors on the primary outcomes. Participants were also instructed to abstain from alcohol, recreational drug use, and activities involving head impacts during this period. Lastly, the study employed a targeted metabolomics approach focused on TCA cycle intermediates. This approach excluded other potentially relevant metabolic factors, such as amino acid profiles, fatty acids concentrations, and endocannabinoid levels, which could have provided further context for interpreting the data.

## Resource availability

### Lead contact

Further information and requests for resources should be directed to and will be fulfilled by the lead contact, Keisuke Kawata (kkawata@iu.edu).

### Materials availability

This study did not generate new unique reagents.

### Data and code availability

• Data: All data reported in this paper will be shared by the lead contact upon request.
• Code: This paper does not report original code.
• Study protocol registration in the ClinicalTrials.gov (ID: NCT04880304): https://clinicaltrials.gov/study/NCT04880304.

## Acknowledgments

The authors would like to thank the participants for their effort, time, and engagement in this study. This work were supported by the 10.13039/100000065National Institute of Neurological Disorders and Stroke (to K.K. and S.D.N.: 1R21NS116548). The mass spectrometer used in this study was provided by a Shared Instrumentation Grant 10.13039/100000002NIH S10RR027822 to S.B. Sponsors had no role in the design or execution of the study; collection, management, analysis, or interpretation of the data; preparation, review, or approval of the manuscript; or decision to submit the manuscript for publication.

## Author contributions

Dr. K.K. had full access to all data in the study and takes full responsibility for the integrity of the data and the accuracy of the data analysis. *Study concept and design*: K.K., M.K.N., S.D.N., W.G.K., and P.D.Q.; *Acquisition of data*: M.K.N., O.O., and L.M.K.; *Experiments, analysis and interpretation of data*: G.E., S.B., G.O.R., K.K., and S.D.N.; *Drafting of the manuscript*: G.E., K.K., and G.O.R.; *Critical revision of the manuscript for important intellectual content*: M.K.N., S.D.N., S.B., W.G.K., G.E., G.O.R., K.K., P.D.Q., O.O., and L.M.K.; *Obtained funding*: K.K. and S.D.N.; *Administrative*, *technical*, *or material support*: K.K., S.B., and W.G.K.; *Study supervision*: K.K., S.D.N., W.G.K., S.B., and M.K.N.

## Declaration of interests

The authors declare no competing interests.

## STAR★Methods

### Key resources table

**Table:** 

REAGENT or RESOURCE	SOURCE	IDENTIFIER
**Biological samples**		

Human venous serum	Peripheral human blood obtained by Kawata’s research team	N/A

**Critical in-house assay**		

Liquid chromatography-mass spectrometry	Barnes lab	N/A

**Software and algorithms**		

R version 3.4.1 with the package nlme	The R Project for Statistical Computing	N/A
Prism 9 (version 9.0.1)	GraphPad Software	N/A

**Other**		

Triaxial accelerometer - GForce Tracker	GForce Tracker Inc	N/A
Trial registration	Clinicaltrials.gov	NCT04641832

### Experimental model and study participant details

#### Participants

From March 2021 through March 2022, potential participants were recruited from local universities and soccer clubs, and 87 participants were screened to be assigned into one of two groups: (1) individuals clinically diagnosed and medicated for ADHD (ADHD group) and 2) age- and sex-matched individuals without ADHD diagnosis (non-ADHD group). The inclusion criteria across both groups were between the ages of 18 and 26 and having at least 5 years of soccer heading experience. Participants in the ADHD group were also required to take their ADHD medication at least 5 days of the week. The non-ADHD group had no history of taking ADHD medication or a prior ADHD diagnosis. The exclusion criteria across both groups included a history of head or neck injury, including concussion, within 3 months prior to the start of the study, a history of learning disabilities (e.g., dyslexia, processing deficits), or major mental disorders (e.g., schizophrenia, bipolar disorder, autism spectrum disorder) other than ADHD. Participants with a history of anxiety or depression were included in the study, as long as symptoms were not quantified as severe in their psychiatric assessment or by their provided psychological diagnostic reports (see sections below). We chose this criterion prior to recruitment in order to maintain generalizability, as anxiety and depression have an increased prevalence rate among college aged students, athletes, and as a result of the COVID-19 pandemic.^53^ The sample size was determined based on previous studies indicating head impact effects on mitochondrial-energy metabolites, where 25 subject per group were estimated to yield a statistical power of at least 0.80 with a significance level set at α = 0.05.

#### Trial design

Once participants met inclusion criteria and were free of exclusion criteria, they completed a self-report mental health questionnaire to obtain demographic information, head impact history, and mental health conditions. Blood samples were collected at three time points: pre-heading baseline, 2 hour-, and 24 hour-post heading. Following baseline measurements, each participant performed 10 controlled soccer headers. Participants then remained in the laboratory until the 2 hour-post heading time point. Participants returned to the laboratory approximately 24 h after the soccer heading for the final time point. All data collection sessions took place during standard daytime hours, avoiding early morning or late evening sessions. Participants also returned to the laboratory for 24 hour-post heading data collection at a time comparable to their baseline sessions. From three days prior to participation in the study and continuing until after the 24 hours-post heading, participants were instructed to: 1) take their prescribed ADHD medication; 2) refrain from consuming alcohol and recreational drugs; 3) refrain from activities that involve head impacts. If participants were unable to follow these instructions, participants were either rescheduled or withdrawn from the study. The study protocol was approved by the Indiana University Institutional Review Board and was registered under ClinicalTrials.gov (NCT04880304). All participants provided written informed consent.

### Method details

#### Diagnostic assessment for ADHD and screening for psychiatric factors

To confirm ADHD diagnosis and assess ADHD symptoms, a well-trained research coordinator, under the guidance of two licensed psychologists with expertise in ADHD, conducted a semi-structured diagnostic interview using the Adult ADHD Investigator Symptom Rating Scale (AISRS).^54^ Two trained-testers assessed the agreement of AISRS total symptom scores, which yielded an excellent inter-rater reliability (intraclass correlation coefficient, 0.99 [95% CI, 0.97 to 1.00]; *p* < 0.001). The coordinator and testers were blinded to the participant group assignment (ADHD vs. non-ADHD). In compliance with the AISRS, participants in the ADHD group were required to report a minimum of five symptoms of either the hyperactive/impulsive or inattentive domain and were free of symptoms unrelated to ADHD, as set forth by the Diagnostic and Statistical Manual of Mental Disorders 5th edition (DSM-5). Documentation of a complete clinical diagnosis of ADHD from participants’ medical provider was also used to further validate inclusion into the ADHD group. Participants with no history of ADHD diagnosis and scored below the AISRS threshold were included in the non-ADHD group. Additionally, this study implemented the following psychological assessments: Beck Anxiety Inventory (BAI),^55^ Patient Health Questionnaire (PHQ-9),^56^ General Anxiety Disorder (GAD-7), Alcohol Use Disorder Identification Test (AUDIT),^57^ Cannabis Use Disorder Identification Test (CUDIT),^58^ and Daily Stress Inventory (DSI).^59^

#### Subconcussion intervention

A standardized and reliable soccer heading protocol was used as a means to induce subconcussive head impacts.^9^^,^^10^^,^^60^^,^^61^^,^^62^^,^^63^ See Bevilaqua et al.^21^ for a video version of the soccer heading protocol. Linear and rotational head accelerations were measured by a triaxial accelerometer (GFroceTracker Inc., Ontario CA). A JUGS soccer machine (JUGS Sports, Tualatin, OR) was used to launch a size-5 soccer ball at a velocity of 25 mph (11.2 m/s). This speed was chosen due to its similar speed to a throw-in or the slower, rising balls kicked by adult soccer players.^64^ Participants in both groups stood 40 feet away from the JUGS machine. Participants were instructed to use the standard method of hitting the ball with the center of their forehead to perform 10 headers within 1-min intervals to a targeted individual who stood about 16 feet (4.9 meters) from participants.

#### LC-MS analysis

Six milliliters of venous blood was collected into vacutainer tubes (BD Biosciences, San Jose, CA) at pre (baseline), 2 hours-post, and 24 hours-post heading. After being allowed to clot, blood samples were centrifuged (1,500 x g for 15 min at 4°C). Serum samples were aliquoted and stored at −80°C. Samples were sent on dry ice to the Targeted Metabolomics and Proteomics Laboratory at the University of Alabama at Birmingham where targeted LC-MS analysis was performed on the TCA cycle intermediates; pyruvate, citrate, isocitrate, alpha-ketoglutarate, succinate, fumarate, malate, oxaloacetate, 2-hydroxyglutarate (2-HG), and methylmalonate.

Sera (100 μL each) were treated with four volumes of ice-cold methanol to precipitate serum proteins and then centrifuged (1,500 x g for 15 min at 4°C). The supernatants were aspirated and taken to dryness under a stream of N_2_ gas. Standards and the dried serum extracts were taken up in 50% aqueous methanol. ^13^C_4_-Succinate (10 μL, 50 ng) was added to each and reacted with O-benzylhydroxylamine (O-BHA) in the presence of 1-ethyl-3-(3-dimethylaminopropyl) carbodiimide (EDC). After a double extraction of the reaction mixture by ethyl acetate to recover the O-BHA conjugates of the metabolic intermediates, the combined ethyl acetate fractions were evaporated to dryness under N_2_.

After dispersal in 100 μL 0.1% formic acid, an aliquot (10 μL) of each sample was loaded onto a 100 mm × 2.1 mm ID, 2.6 μm Accucore reverse-phase column (Thermo Fisher, Waltham, MA). Chromatography was carried out at 40°C using the following mobile phases: A – ddH_2_O with 0.1% formic acid; B – acetonitrile/0.1% formic acid. Columns were initially equilibrated with solvent containing 10% B. After injection of the samples, the metabolites were eluted with a linear gradient to 30% B by 1 min, by a linear gradient to 85% B at 5.5 min, by a linear gradient to 95% by 5.6 min and a hold at 95% B to 6.5 min. The column was then re-equilibrated for 2 min with 10% B. A Prominence HPLC (Shimadzu) provided a flow rate of 500 μL/min. An SCIEX 4000 triple quadrupole mass-spectrometer (Toronto, Ontario) operating in the positive mode was used to analyze TCA metabolites. IonSpray voltage for positive mode was +5000. Declustering potential was +80 V. IonSpray GS1/GS2 were set at 50 psi, with curtain gas set at 30 psi. Interface heater temperature was set at 500°C. The mass spectrometer was operated in the multiple reaction mode – the following mass transitions were observed – pyruvate *m/z* 299 ->181, citrate *m/z* 508-> 385, isocitrate *m/z* 508->385, 2-hydroxyglutarate *m/z* 359->236, alpha-ketoglutarate *m/z* 462-> 339, succinate *m/z* 329-> 206, ^13^C-succinate internal standard *m/z* 333->210, fumarate *m/z* 327-> 204, malate *m/z* 345-> 222, and oxaloacetate *m/z* 448->325. Pooled samples, containing 20 μL of each specimen, were injected into every tenth analysis to assess instrument stability with the data collection protocol. MultiQuant version 3.0.3 was used for post-acquisition data analysis. Peak areas of the TCA intermediates and standards were normalized by the area of the ^13^C_4_-succinate peak.

### Quantification and statistical analysis

#### Statistical analyses

Demographic differences between the ADHD and non-ADHD groups were assessed by two-tailed independent samples t-tests for continuous variables (age, BMI, soccer experience, soccer heading experience, previous number of concussions, concussion-related symptoms at baseline, AUDIT score, and CUDIT score) and chi-squares for categorical variables (sex, race, ethnicity, and additional mental health diagnoses).

To examine the within-group and between-group pattern of mitochondrial responses to head impacts, as reflected in blood metabolite profiles of the TCA cycle, a series of multivariable mixed-effect regression models (MRMs) was conducted with the 10 metabolites as outcome variables. The primary (fixed-effect) factors were groups (ADHD vs. non-ADHD), timepoint (pre-heading baseline and 2 hours- and 24 hours-post heading), and the group-by-time interaction. The group differences were obtained by using a group-by-time interaction. Participants were treated as the random effect to account for individual differences at baseline. The model accounted for the repeated measures from the same participants and included the potential covariates of number of previous concussion and mental health diagnosis (dummy coded as 0 = no diagnosis, 1 = depression, 2 = anxiety, 3 = both). Analysis for each model was detailed in the results section with a format of a contrast estimate with its 95% confidence interval (CI) and *p*-value in the following format: (b estimate [95% CI, low CI-high CI], *p*-value). The significance levels for all tests were set to 0.05. A post-hoc analysis using marginal means was conducted to examine group differences in metabolite level at each of the three timepoints, and a Bonferroni adjusted alpha level of 0.017 was considered statistically significant. All analyses were conducted using R, version 4.4.2 (R Project for Statistical Computing) using packages nlme and emmeans.
