# Supplementary material for: Alterations in mitochondrial energy metabolites following acute subconcussive head impacts among athletes with and without ADHD
Source: iScience. 2025 May 28;28(6):112776. doi: 10.1016/j.isci.2025.112776 (PMC12192527; doi:10.1016/j.isci.2025.112776)
Supplement: Document S1. Table S1 [file mmc1.pdf]

iScience, Volume *28*

Supplemental information

Alterations in mitochondrial energy metabolites following acute subconcussive head impacts among athletes with and without ADHD

Gage Ellis, Madeleine K. Nowak, William G. Kronenberger, Grace O. Recht, Osamudiamen Ogbeide, Lillian M. Klemsz, Patrick D. Quinn, Landon Wilson, Taylor Berryhill, Stephen Barnes, Sharlene D. Newman, and Keisuke Kawata

Supplemental Table 1: Group average of metabolite values at each timepoint				
Metabolites	Group	T1<br>Pre-Heading	T2<br>2-Hours Post-Heading	T3<br>24-Hours Post-Heading
Pyruvate (µg/mL)	Overall	509.34 ± 308.89	680.23 ± 472.94	823.83 ± 506.38
	ADHD	502.46 ± 266.78	638.81 ± 242.02	686.79 ± 380.64
	Control	516.22 ± 351.47	721.65 ± 628.11	960.87 ± 582.67
Citrate (µg/mL)	Overall	66.99 ± 43.87	37.07 ± 29.26	12.18 ± 17.80
	ADHD	86.07 ± 46.16	40.48 ± 30.45	17.63 ± 22.60
	Control	47.91 ± 32.24	33.67 ± 28.21	6.73 ± 8.63
Isocitrate (µg/mL)	Overall	2.63 ± 1.48	1.84 ± 1.22	0.68 ± 0.66
	ADHD	3.26 ± 1.32	2.00 ± 1.21	0.83 ± 0.81
	Control	2.00 ± 1.39	1.68 ± 1.23	0.53 ± 0.42
Alpha-Ketoglutarate (µg/mL)	Overall	10.75 ± 4.43	12.00 ± 6.45	16.78 ± 6.98
	ADHD	11.61 ± 4.61	10.94 ± 4.55	13.87 ± 4.42
	Control	9.88 ± 4.15	13.06 ± 7.86	19.68 ± 7.90
Succinate (µg/mL)	Overall	12.72 ± 3.06	13.61 ± 3.04	13.95 ± 3.54
	ADHD	13.31 ± 2.93	13.10 ± 3.47	14.08 ± 3.53
	Control	12.13 ± 3.13	14.13 ± 2.49	13.82 ± 3.62
Fumarate (µg/mL)	Overall	1.73 ± 1.09	1.99 ± 1.37	3.03 ± 1.50
	ADHD	1.84 ± 1.06	1.84 ± 1.06	2.64 ± 1.08
	Control	1.63 ± 1.14	2.14 ± 1.63	3.41 ± 1.77
Malate (µg/mL)	Overall	10.80 ± 4.47	9.53 ± 2.47	8.21 ± 3.43
	ADHD	11.87 ± 4.97	9.91 ± 2.34	8.85 ± 3.65
	Control	9.73 ± 3.70	9.16 ± 2.58	7.58 ± 3.13
Oxaloacetate (µg/mL)	Overall	1.53 ± 1.54	1.10 ± 0.84	0.72 ± 1.42
	ADHD	2.12 ± 1.97	0.94 ± 0.80	0.89 ± 1.8
	Control	0.95 ± 0.48	1.26 ± 0.87	0.56 ± 0.89
2-HG (µg/mL)	Overall	1.20 ± 0.55	1.41 ± 0.87	2.00 ± 0.83
	ADHD	1.26 ± 0.55	1.39 ± 0.75	1.76 ± 0.74
	Control	1.15 ± 0.56	1.44 ± 1.00	2.24 ± 0.86
Methylmalonate (µg/mL)	Overall	0.87 ± 0.37	0.91 ± 0.35	1.18 ± 0.44
	ADHD	0.88 ± 0.31	0.86 ± 0.27	1.04 ± 0.37

	Control	0.86 ± 0.44	0.96 ± 0.42	1.32 ± 0.46
**Note:** Mean ± standard deviation				
